# Pulmonary Hypertension Due to Common Respiratory Conditions: Classification, Evaluation and Management Strategies

**DOI:** 10.3390/jcm5090075

**Published:** 2016-08-26

**Authors:** Daniel G. Fein, Ali N. Zaidi, Roxana Sulica

**Affiliations:** 1 Division of Pulmonary Medicine, Montefiore Medical Center, 111 East 210th Street, Bronx, NY 10467, USA; 2 Division of Cardiology, Montefiore Medical Center, 111 East 210th Street, Bronx, NY 10467, USA; azaidi@montefiore.org; 3 Division of Pulmonary Critical Care and Sleep Medicine, Mount Sinai Beth Israel, 7 Dazian Building 1st Avenue at 16th Street, New York, NY 10003, USA; rsulica@chpnet.org

**Keywords:** lung diseases, Interstitial Pulmonary Disease, Chronic Obstructive Sleep Apnea Syndromes

## Abstract

Pulmonary hypertension (PH) due to chronic respiratory disease and/or hypoxia is classified as World Health Organization (WHO) Group III pulmonary hypertension. The patients most commonly encountered in clinical practice with group III PH include those with chronic obstructive lung disease (COPD), diffuse parenchymal lung disease, and sleep-disordered breathing. The purpose of this review is to outline the variable clinical significance of pulmonary hypertension in the most common pulmonary disease states and how a clinician may approach the management of these patients.

## 1. Introduction

The use of medications to vasodilate and remodel the pulmonary vasculature has been shown to improve morbidity and even mortality in patients who suffer from pulmonary arterial hypertension (PAH) [1,2], also known as group I pulmonary hypertension PH, which is a rare disorder. In contrast, PH secondary to heart (group II) or lung disease (group III) is much more frequently encountered by clinicians, but these forms of PH are less well studied and lack effective approved therapies. Group III PH may be due to a wide variety of common and less common pulmonary conditions (Table 1). The purpose of this article is to review the pathophysiology and clinical presentation of patients with PH secondary to common lung diseases and to identify available or innovative approaches to the management of this commonly encountered disease.

## 2. Classification of Group III PH

In 1998, at the second World Symposium on pulmonary hypertension held in Evian, France, pulmonary hypertension was divided into five groups in order to better define pathologic associations and streamline treatment strategies [1]. Group III PH is currently classified by right heart catheterization (RHC) findings of pre-capillary PH (mean pulmonary artery pressure (mPAP) >25 mmHg, pulmonary artery wedge pressure (PAWP) ≤ 15 mmHg with normal or reduced cardiac output) secondary to chronic obstructive pulmonary disease (COPD), interstitial lung disease (ILD), other pulmonary diseases with mixed restrictive and obstructive patterns, sleep-disordered breathing, alveolar hypoventilation disorders, chronic hypoxia or developmental abnormalities of the lung [3].

It has been suggested that the term “out of proportion” be abandoned when classifying patients with pulmonary hypertension in the setting of chronic obstructive pulmonary disease (COPD), idiopathic pulmonary fibrosis (IPF) or combined pulmonary fibrosis with emphysema (CPFE). Instead, it has been proposed that the respective pulmonary disease be classified as without PH, with PH or with severe PH [4]. Patients with intrinsic lung diseases may be classified as without PH if the mPAP as measured by RHC is less than 25 mmHg and with PH if the mPAP is greater than or equal to 25 mmHg. If the mPAP is greater than or equal to 35 mmHg or the mPAP is greater than 25 mmHg with a low cardiac index (CI < 2.0 L/min/m^2^), then patients are delineated as severe PH-COPD/IPF/CPFE (e.g., severe PH-COPD).

## 3. Pathophysiology

Pulmonary hypertension in the setting of chronic respiratory disease leads to increased pulmonary vascular resistance (PVR) when the PAWP is normal. Increased PVR is usually secondary to the effects of hypoxia and destruction of the vascular bed in emphysematous or fibrotic areas of lung parenchyma. While acute hypoxia leads to pulmonary vasoconstriction in the small pre-capillary arteries, chronic hypoxemia results in pulmonary vascular remodeling including medial hypertrophy and intimal obstructive proliferation of the distal pulmonary arteries [5]. Additional mitigating factors may include mechanical stress secondary to hyperinflation, loss of capillaries, inflammation, cigarette smoke toxicity and endothelium-derived vasoconstrictor-vasodilator imbalance [3]. Moreover, in a significant number of patients with lung disease and severe PH, other co-morbidities may contribute to the pathogenesis of PH, such as left ventricular diastolic dysfunction or chronic thromboembolic pulmonary hypertension. Genetic factors may also mediate the underlying risk of some patients for developing PH. Gene polymorphisms of serotonin transporters which play a role in pulmonary artery smooth muscle hyperplasia have been found to be associated with the severity of PH in hypoxemic patients with COPD [6].

## 4. Diagnosis of Group III PH

The recognition and diagnosis of PH may be challenging due to the presence of coexisting symptomatology in patients with advanced respiratory disease. Dyspnea on exertion is often common in patients with underlying lung disease regardless of the presence of PH. The relatively mild degree of PH in most of the patients with pulmonary disease prevents the many objective diagnostic characteristics that are helpful to identify patients with PAH, such as a loud pulmonary component to the second heart sound, a pan-systolic murmur of tricuspid regurgitation, and EKG or chest X-ray findings, from being useful until patients have advanced right heart failure [7]. It has been suggested that a further work-up for PH be undertaken in the presence of pulmonary disease when dyspnea is deemed out of proportion to objective measures of illness. Clinicians may also consider that in patients with COPD, severe hypoxemia, hypocapnea, mild to moderate airway obstruction and very low diffusing capacity have been subsequently associated with severe PH as measured by RHC [8]. 

Should a clinician choose to proceed with further initial evaluation of suspected PH in the setting of lung disease, the diagnostic algorithm is similar to that of all patients with suspected PH. A general strategy that has been proposed includes the measurement of diffusing capacity and static lung volumes followed by CT imaging and transthoracic echocardiography (TTE) [7]. The diagnostic accuracy of TTE to measure pulmonary artery pressure in patients with lung disease has been found to be frequently inaccurate and may lead to the over-diagnosis of PH in up to half of all patients examined [9]. Despite this limitation, TTE is recommended for the non-invasive assessment of all patients with lung disease and suspected PH [10].

The measurement of biomarkers, particularly brain natriuretic peptide (BNP), has been suggested as a useful preliminary test for both evaluation and risk stratification of patients with chronic lung disease and suspected PH. Leuchte et al. found that elevated BNP concentrations identified significant pulmonary hypertension with high sensitivity and specificity and elevated levels were found to be highly predictive of patient mortality [11]. As a result of these findings, BNP is considered useful to identify patients with severe disease and to risk-stratify patients who may be at particular risk of adverse outcomes.

Although RHC is the gold standard for the diagnosis all classes of PH, it is suggested that RHC be performed for the diagnosis of group III PH in only select circumstances. These scenarios include suspicion of severe PH (TTE right ventricular systolic pressure greater than 50 to 60 mmHg and/or the presence of right heart abnormalities), the opportunity for findings that impact management (referral for lung volume reduction surgery or transplant), lack of explanation of patient symptomatology by existing objective testing (pulmonary function studies, CT of the chest), or consideration for the initiation of PH-directed therapy or indication of right heart failure (Table 2) [12]. Additionally, RHC may also be indicated to rule out left-sided heart disease as a mediator of symptoms or right-sided heart dysfunction. Our practice mirrors guideline-based recommendations to perform RHC only when therapeutic consequences are anticipated. These may include evaluation for lung transplantation, exploration of an alternative diagnosis (PAH) or if the patient is being evaluated for a clinical trial [10].

In summary, the diagnosis of PH in patients with lung disease is challenging. A high level of clinical suspicion due to out-of-proportion symptoms and severe gas exchange abnormalities should be followed by pulmonary function testing and radiologic investigation such as high resolution CT scan and V/Q scan. The work-up may continue with further risk stratification using measurements of biomarkers as well as TTE and be completed with confirmatory RHC in select cases.

## 5. Survival in Group III PH

The presence of pulmonary hypertension in patients with lung disease is associated with a significant negative impact on survival. The prognostic implication, however, varies between disease states. In a retrospective review of 178 patients with group III disease in the ASPIRE registry, the overall three-year survival was 44%. Among their examined cohort, the patients with sleep-disordered breathing or alveolar hypoventilation (25 patients) had the best three-year survival (90%), PH associated with interstitial lung disease (32 patients) had the worst survival at 16% and COPD-associated PH (101 patients) was noted to have intermediate prognosis as compared to the other disease states (41% three-year survival) [13].

## 6. Group III Pulmonary Hypertension in Specific Disease States

### 6.1. Chronic Obstructive Pulmonary Disease (COPD)

Chronic obstructive pulmonary disease (COPD) is a common condition that is thought to affect 10% of the world’s population [14]. Estimations of the prevalence of PH in COPD vary according to the population studied, the diagnostic methods, and the definition of PH used. Many patients included in observational studies are those awaiting transplantation and therefore are representative of a cohort with more advanced lung disease, leading to a an overstated prevalence estimate. Andersen et al. reported on retrospective data from 409 patients with COPD in the Scandia Transplant Registry and found that 146 patients (35.7%) had elevated mPAP with a pulmonary artery wedge pressure less than or equal to 15 mmHg, thus designating them as having PH [15].

In similar retrospective, observational studies of patients under evaluation for lung transplant, the incidence of PH has been noted to be between 23% and 50% with only a minority noted to have mPAP ≥ 35 mmHg [16,17]. This mild to moderate elevation of pulmonary pressures is thought to be characteristic of the hemodynamic findings in PH associated with lung diseases and/or hypoxemia.

The presence of PH in COPD has also been associated with a reduced 6 min walk distance (6MWD) [15,18], with one study of 362 patients showing that for every increase of the mPAP by 5 mmHg, the 6MWD decreased by 11 m [17]. In the subset of patients with COPD who have severe PH, Chaouat et al. found that increased dyspnea on exertion, severe hypoxemia and hyperventilation characterized their presentation. Additional common clinical characteristics included mild to moderate airway obstruction, minimally decreased forced expiratory volume in 1 second (FEV1) and very low diffusing capacity [8].

There is extensive literature citing the presence of PH as a poor prognostic marker in COPD [8,18,19,20,21]. PH was first described as a marker of poor prognosis among patients with COPD in 1972; among 50 patients evaluated with RHC, Burrows et al. demonstrated that increased PVR was the factor most strongly associated with reduced survival [19]. In this cohort of patients with COPD who underwent evaluation for lung transplant, the five-year survival was substantially lower in those with PH (37%) than in patients who did not have PH (63%). Overall, PH secondary to COPD is a common phenomenon. Severe elevations of the mPAP found on RHC are associated with worse survival as compared to patients with COPD and mild hemodynamic disease.

### 6.2. Idiopathic Pulmonary Fibrosis

PH may develop in patients with a variety of rare, diffuse lung diseases such as sarcoidosis, pulmonary Langerhans cell histiocytosis and lymphangioleiomyomatosis. These disease states are systemic in nature and are classified as group V PH, with an unclear and/or multifactorial mechanism. In clinical practice, idiopathic pulmonary fibrosis (IPF) is one of the most frequently encountered diffuse lung diseases and will therefore be the focus of this review. As with other causes of PH due to lung disease, the development of PH in IPF is thought to be at least partly due to hypoxemia-induced vascular remodeling. Additional contributing processes with deleterious effects include IPF-specific hyperplasia and fibrosis of the elastic lamina of small pulmonary arteries, in situ thrombosis in small pulmonary arteries as well as intimal proliferation and fibrosis of the pulmonary venules. Multiple IPF-mediated cytokines, including endothelin-1, serotonin, platelet-derived growth factor and transforming growth factor beta, are also thought to contribute to PH associated with IPF [22]. Similarly to estimates of COPD, the prevalence of PH in IPF is variably reported across different study populations, and depends on methods of evaluation and criteria for diagnosis. TTE-estimated PH has been found in between 32% and 84% of patients with IPF [23,24,25], with right ventricular dysfunction noted in 50 out of 77 patients in one cohort [23] and increased mean mPAP correlated with reduced diffusing capacity for carbon monoxide [24,25]. Much like when PH is found to co-exist with COPD, elevated mPAP has been found to be an independent predictor of survival in patients with IPF. One-year survival has been shown to be reduced from 28% to 5.5% when compared to patients without PH awaiting lung transplantation [26].

### 6.3. Combined Pulmonary Fibrosis and Emphysema

Combined pulmonary fibrosis and emphysema (CPFE) is defined by CT findings of emphysema in the upper lung zones with basal or peripheral lung fibrosis characterized as reticular opacities, honeycombing, architectural distortion and/or traction bronchiectasis. Patients with CPFE have been found to have a high prevalence of pulmonary hypertension and worse survival when compared to patients with IPF and no emphysema [27,28]. Lower diffusing capacity, higher PVR and lower CI are all associated with shorter survival in this patient population [28]. Clinicians should be aware that CPFE is often associated with PH, and that PH in patients with CPFE is associated with worse outcomes. 

### 6.4. Obstructive Sleep Apnea

Obstructive sleep apnea (OSA) is defined by daytime sleepiness, snoring and witnessed breathing interruptions or awakenings due to gasping or choking in the presence of at least five obstructive respiratory events per hour of sleep [29]. PH associated with OSA is a common but poorly understood phenomenon. PH in patients with OSA is thought to largely be due to hypoxia-related vasoconstriction, which occurs during apneic episodes, which may lead to subsequent vascular remodeling. Hypercapnea, alterations in cardiac physiology, adverse effects of negative intra-thoracic pressure, sympathetic dysfunction, and obesity-related pulmonary vascular remodeling have also been hypothesized to be important in the development of adverse pulmonary vascular physiology and right ventricular dysfunction [30]. The high rate of co-existent diastolic left heart failure in patients who suffer from OSA additionally complicates the underlying physiologic basis for this condition [31].

Similarly to other etiologies of group III PH, the estimations of the prevalence of PH associated with sleep-disordered breathing are limited by varying study methodologies and disparate criteria. Using TTE, Sajdov et al. noted PH in 47% of patients with OSA [32]; while using RHC, Bady et al. diagnosed PH in 27% of patients with OSA [33]. The degree of pulmonary hypertension was noted to be moderate (mean mPAP 28.5 mmHg). Patients with OSA who have PH are more likely to be obese, have lower FEV1, vital capacity (VC) and FEV1/VC ratio as well as lower PaO_2_ and higher PaCO_2_ [34].

In patients with OSA and PH, continuous positive airway pressure (CPAP) therapy has been found to be efficacious for lowering pulmonary pressures as measured by TTE [35,36]. Despite this evidence, the clinical significance of these findings is uncertain. Further, there is no known effective pharmacotherapy for PH associated with OSA. Oxygen supplementation is often employed, particularly if patients are intolerant of CPAP therapy, but the efficacy of this strategy to reduced PH has not been demonstrated. 

OSA has been shown to be an independent causal factor for the devolvement of PH. PH, when found in patients with OSA, is usually a more benign prognostic marker [13], compared to other group III forms of PH. PH secondary to OSA has been shown to be somewhat reversible with effective CPAP therapy but the benefits of supplemental oxygen have not been demonstrated.

## 7. Treatment Strategies for Group III PH

In general, the treatment of pulmonary hypertension can be divided into primary and advanced therapies. Primary therapies target the underlying disease process, while advanced therapeutics seek to mitigate the pulmonary vascular disease itself through the alteration of cellular proliferation, vasoconstriction and vascular remodeling. The first step in the primary management of group III pulmonary hypertension involves optimization of the underlying lung disease according to relevant clinical practice guidelines [37].

### 7.1. Primary Therapy

#### 7.1.1. Oxygen

An important standardized step in the treatment of group III PH is the administration of supplemental oxygen if indicated. A mortality benefit has been demonstrated among patients with COPD and hypoxemia by two separate investigations [38,39]. More specifically, the effect of oxygen supplementation on pulmonary hemodynamics was reported on by Weitzenblum et al. who found a progressive increase in mPAPs prior to supplemental oxygen administration followed by a decrease after substantial time on oxygen therapy [40]. Although these changes suggested that long-term oxygen therapy in patients with severe COPD could mitigate the progression of PH in many patients, it must be noted that complete pulmonary artery pressure normalization was rarely observed. In accordance with guideline-based recommendations we suggest the addition of supplemental oxygen for patients with COPD when arterial blood oxygen pressure is less than 8 kPa (60 mmHg) or arterial oxygen saturation is below 91%. Supplemental oxygen may be prescribed on exertion when there is an indication of correction of hypoxemia which coincides with symptom relief [10].

#### 7.1.2. Diuretics

Diuretics are frequently employed to mediate intravascular volume in patients with group III PH who have fluid retention secondary to right ventricular failure [3]. Although there are no randomized trials to support this practice, empiric clinical experience suggests that patients receive symptomatic improvement following diuresis in the setting of decompensated right heart failure. Patients undergoing fluid removal via diuretics should be monitored for electrolyte derangements and pre-renal failure. The practice of administering diuretics to patients with PH is supported by guideline-based recommendations [10].

#### 7.1.3. Anticoagulation

The use of anticoagulation in patients with idiopathic pulmonary artery hypertension (PAH), heritable PAH and PAH due to anorexigens has been generally recommended in expert consensus guidelines [3,41] based on a high incidence of vascular thrombotic lesions on post-mortem examination of patients with idiopathic PAH and reports of abnormal coagulation and fibrinolytic pathways in this patient population [42]. Recently published retrospective data has been inconsistent in demonstrating a mortality benefit of anticoagulation in patients with PAH [43,44]. As such, the use of routine anticoagulation for patients with group III PH is not recommended unless an alternative patient-specific indication for anticoagulation exists.

#### 7.1.4. Vaccinations 

Pneumonia is thought to be implicated in up to 7% of deaths of patients with PAH [3]. For this reason, vaccination against influenza and pneumococcal pneumonia is generally recommended for all groups of patients with PH including those with PH related to underlying lung disease. These recommendations encompass expert opinion including Centers for Disease Control and Prevention (CDC) guidance regarding patients with COPD and other advanced lung diseases [45].

#### 7.1.5. Pulmonary Rehabilitation

Pulmonary rehabilitation is recognized as a potentially important component of the treatment plan for a wide spectrum of chronic respiratory conditions. Most patients currently enrolled in formalized exercise programs have a diagnosis of COPD; however, there is data to suggest that individuals with a variety of other respiratory diseases may benefit from this treatment modality [46]. Studies of the impact of pulmonary rehabilitation on patients with PH have suggested an association between exercise training in pulmonary hypertension and improved skeletal muscle function, quality of life and exercise capacity [47,48,49,50]. These findings may be applied to patients with pulmonary hypertension due to lung diseases or hypoxemia and we suggest a referral to pulmonary rehabilitation when feasible. 

### 7.2. Targeted PH Therapy

Based on current evidence, treatment of PH due to chronic respiratory conditions centers on the optimization of the management of the underlying lung disease. PAH-specific drug therapy is not recommended for patients with PH secondary to lung diseases due to both physiologic concerns and the lack of quality data to support the use of these drugs in this patient population [3,10].

Physiologically, pulmonary vasodilators such as nifedipine and nitric oxide have been demonstrated to inhibit hypoxic pulmonary vasoconstriction in patients with COPD and thus adversely affect gas exchange by worsening ventilation to perfusion mismatching [51,52]. Most investigations into PH-targeted therapy have shown conflicting results and are limited by study design and small sample size. 

While some investigations into sildenafil administration in patients with PH secondary to lung fibrosis or COPD have demonstrated improved gas exchange [53], hemodynamics [54], and 6 min walk test [53,55,56], others have failed to show improved stroke volume or increased exercise capacity [57]. The impact of endothelin receptor antagonists on patients with PH due to lung disease has also been investigated. The impact of bosentan on COPD-associated PH has been studied in at least two trials, with controversial results. In one randomized trial, not only did bosentan not improve exercise capacity, it also led to worsened oxygenation, and quality of life [58]. In another controlled trial, with more selective enrollment criteria, patients with severe PH (mean mPAP 37 mmHg, all patients diagnosed by RHC) benefited from bosentan administration as measured by improved exercise capacity and hemodynamics [59]. Other studies of the effect of bosentan on PH related to IPF have failed to demonstrate improvements in gas exchange [60], hemodynamics, functional capacity or symptoms [61]. In patients with IPF, ambrisentan has been found to be associated with disease progression as defined by death, respiratory hospitalization or decreased lung function and is therefore contraindicated in this subset of patients [62]. 

Despite some positive signals evident in the above-noted investigations, results are limited by flawed study design, including small sample sizes, lack of randomization and lack of controls. This weakly supported evidence impedes the translation of findings into readily applicable practice. For this reason, the above therapies may be regarded as experimental until more robust data becomes available and, by extension, targeted therapy in patients with group III PH is not currently universally recommended outside of clinical trials in specialized centers with expert consultation. We do not support the use of targeted therapy outside of patients who have indication of PAH as evaluated by RHC. 

## 8. Conclusions

Group III PH is defined as PH caused by chronic lung disease and/or hypoxia. It is most commonly associated in clinical practice with COPD, parenchymal lung disease and sleep-disordered breathing. Although pulmonary hemodynamics in this patient population are often not significantly abnormal, the presence of PH correlates with worsened functional impairment and is a harbinger of poor outcomes (Table 3). The fundamental approach to care involves the optimization of the underlying pulmonary disease, oxygen therapy, pulmonary rehabilitation, diuretics and appropriate vaccinations. For the present time, targeted therapy should be utilized only with the guidance of expert consultation.

## Figures and Tables

**Table 1:** Disease states associated with group III pulmonary hypertension.

Disease States
Chronic Obstructive Pulmonary Disease
Idiopathic Pulmonary Fibrosis
Combined Pulmonary Fibrosis and Emphysema
Sleep Disordered Breathing
Alveolar Hypoventilation Disorders
Chronic Hypoxia
Developmental Abnormalities of the Lung

Adapted from Galiè et al. [3].

**Table 2:** Indications for right heart catheterization in patients with group III PH.

Indications
Severe PH Suspected
Findings may change management (referral for lung volume reduction or transplant)
Patient symptoms cannot be explained by existing objective testing
PH-directed therapy being considered
Indication of right heart failure

Adapted from Minai et al. [11]; PH = pulmonary hypertension.

**Table 3:** Summary.

Key Points
Group III PH is Generally Associated with Mild to Moderate Elevations in mPAP
The presence of group III PH is correlated with worse functional status and outcomes
Treatment should be aimed at optimization of the underlying pulmonary condition along with oxygen therapy, pulmonary rehabilitation, diuretics and vaccinations
There is limited data to support the use of pulmonary vasodilator therapy for group III PH

PH = pulmonary hypertension; mPAP = mean pulmonary artery pressure.
